# Altered Gene Expression by Low-Dose Arsenic Exposure in Humans and Cultured Cardiomyocytes: Assessment by Real-Time PCR Arrays

**DOI:** 10.3390/ijerph8062090

**Published:** 2011-06-08

**Authors:** Jinyao Mo, Yajuan Xia, Timothy J. Wade, David M. DeMarini, Mercy Davidson, Judy Mumford

**Affiliations:** 1 Center for Environmental Medicine, Asthma and Lung Biology, University of North Carolina, Chapel Hill, NC 27599, USA; E-Mail: jin_mo@med.unc.edu; 2 Inner Mongolia Center for Endemic Disease Control and Research, Huhhot 010031, Inner Mongolia, China; E-Mail: yajxia@126.com; 3 National Health and Environmental Effects Research Laboratory, U.S. Environmental Protection Agency, Research Triangle Park, NC 27711, USA; E-Mails: demarini.david@epa.gov (D.M.D.); jmumford5@gmail.com (J.M.); 4 Department of Radiation Oncology, Columbia University, New York, NY 10032, USA; E-Mail: mmd2@columbia.edu

**Keywords:** arsenic, drinking water, low-density array, real-time PCR, cardiomycocytes

## Abstract

Chronic arsenic exposure results in higher risk of skin, lung, and bladder cancer, as well as cardiovascular disease and diabetes. The purpose of this study was to investigate the effects on expression of selected genes in the blood lymphocytes from 159 people exposed chronically to arsenic in their drinking water using a novel RT-PCR TaqMan low-density array (TLDA). We found that expression of tumor necrosis factor-α (*TNF-α*), which activates both inflammation and NF-κB-dependent survival pathways, was strongly associated with water and urinary arsenic levels. Expression of *KCNA5*, which encodes a potassium ion channel protein, was positively associated with water and toe nail arsenic levels. Expression of 2 and 11 genes were positively associated with nail and urinary arsenic, respectively. Because arsenic exposure has been reported to be associated with long QT intervals and vascular disease in humans, we also used this TLDA for analysis of gene expression in human cardiomyocytes exposed to arsenic *in vitro*. Expression of the ion-channel genes *CACNA1, KCNH2, KCNQ1* and *KCNE1* were down-regulated by 1-μM arsenic. Alteration of some common pathways, including those involved in oxidative stress, inflammatory signaling, and ion-channel function, may underlay the seemingly disparate array of arsenic-associated diseases, such as cancer, cardiovascular disease, and diabetes.

## 1. Introduction

Extensive epidemiological studies have demonstrated that environmental exposure to arsenic causes cancer of the skin, lung, and bladder [1–3] and is also a co-carcinogen in combination with UV light for skin cancer [4]. In addition, exposure to arsenic either chronically through the drinking water [5] or acutely through medical therapy for acute promyelocytic leukemia or poisoning [6,7] is associated with cardiovascular abnormalities [8,9]. This is frequently manifest as a prolongation of the QT (time between initial deflection of QRS complex to the end of T wave) interval as measured on an electrocardiogram, resulting in life-threatening malignant ventricular arrhythmias [7].

Mechanistic studies indicate that arsenic enhances inflammation of vascular tissues, activates oxidative signaling, disrupts lipid metabolism, and increases lipid oxidation [8]. These effects can produce endothelial cell dysfunction directly to the vascular cells and indirectly through the liver. In addition, the epigenetic changes induced by arsenic in the liver appear to produce a chronic state of stress and inflammation, resulting in not only liver disease but also systemic vascular disease [8]. In particular, arsenic exposure increases the risk of prolonged QT interval duration, which is associated with atherosclerosis [10,11]. No studies have implicated a role for arsenic on cardiomyocytes *per se*; however, to our knowledge, no *in vitro* exposures of such cells to arsenic have been performed.

To understand these diverse effects of arsenic, toxicogenomic analyses involving cDNA microarray have been used to identify alterations in global gene expression and pathways in cells exposed to arsenic *in vitro* as well as in animals and humans exposed to arsenic [12–16]. The aberrantly expressed genes identified in these studies include those coding for heat shock proteins, DNA damage response, antioxidant activity, inflammation signaling, cell-cycle regulation, and apoptosis [16].

Although global gene expression studies have provided considerable insights into the toxicity pathways affected by arsenic, real-time PCR (RT-PCR) has been used to confirm the arsenic-associated altered gene expression of only a few genes in these studies. The development of a novel qRT-PCR-based technique called Taqman Low-Density Array (TLDA) allows for the simultaneous testing of 48 or 96 genes in a micro-fluidic card format in which the user can specify the genes to be analyzed [17]. This technology has been shown to be a sensitive and reproducible system for analyzing gene expression [18], and it could be of value in confirming the ability of arsenic to alter the expression of genes identified initially by cDNA microarray.

To explore this possibility, and especially to assess some of the toxicogenomic effects of arsenic associated with cardiovascular abnormalities, we used a TLDA to evaluate gene expression in 48 genes in RNA isolated from (a) blood of 159 subjects exposed chronically to arsenic as well as (b) human cardiomyocytes exposed *in vitro* to arsenic. The 48 genes (Table 1) were selected based on the previous cDNA microarray studies described above and fell into 10 functional groups: endogenous control, Nrf2 pathway, heat shock protein, apoptosis, inflammation, NF-κB pathway, cell proliferation DNA methylation, DNA repair, and ion channel.

This array was used to evaluate gene expression in an arsenic-treated human cardiomyocyte cell line (AC16) and in blood from subjects living in Bayingorman (Ba Men) Inner Mongolia, China. This population has been exposed to a wide range of arsenic levels (from non-detectable to 1.8 mg/L), mainly via drinking water from contaminated artesian wells for more than 20 years [19]. Seafood consumption is not common in this population, and arsenic-containing pesticides have not been used in Ba Men [20]. In Ba Men, more than 300,000 people have been chronically exposed to arsenic, especially in three counties: Hangjin Hou, Lin He and Wu Yuan. Arsenic-associated health effects in this population include cancer, dermal, neurological, cardiovascular, and peripheral vascular diseases [9,19]. More than 80 percent of the families owned individual wells, making it possible to assess the arsenic exposure at the individual level.

We assessed arsenic levels in the water from each household well and also in toenails and urine of each subject. This exposure assessment permitted us to associate gene expression in the blood of the subjects to each measure of exposure. In addition, we compared the results in the blood to those determined in cardiomyocytes exposed to arsenic *in vitro*. This provided an opportunity to see if arsenic might have a direct effect on such cells, resulting in changes in gene expression found in blood and/or consistent with the cardiovascular myopathies associated with arsenic exposure.

## 2. Experimental Section

### 2.1. Study Subjects

The study subjects included a total of 159 non-smoking Ba Men residents from the sub-villages of Wulan, Jianshe, Fengchan, and Xinyao located in Sha Hai Village, Hangjn Hou County and the sub-villages of Miaohao and Xigelian located in Sheng Feng Village, Wu Yuan County.

Questionnaires were administered to all participants to obtain demographic information, arsenic exposure, diet, smoking, occupation, pesticide use, and medical information. This study was conducted according to the recommendations of the World Medical Association Declaration of Helsinki [21] for international health research. All subjects gave written informed consent to participate in this study. The research protocol met the requirements for protection of human subject certification by the U.S. Environmental Protection Agency.

### 2.2. Water Collection and Analysis

Samples of drinking water from wells were collected from all subjects’ homes in acid-washed tubes, transported to the U.S. on blue ice, and analyzed for total arsenic using inductively coupled plasma mass spectrometry (ICPMS) as described previously [22]. The detection limit by ICPMS was 0.1 μg of As^III^/L.

### 2.3. Toenail and Urine Collection and Analysis

Toenail samples were collected from the study subjects and analyzed as described previously [23]. The nail samples were first cleaned by sonication in HPLC-grade water, then acetone was added to remove the organic contaminants from the nail surface. Nail samples were analyzed for arsenic concentration by instrumental neutron activation analysis (INAA) at the Nuclear Services Department, North Carolina State University, Raleigh, NC, USA [24]. The detection limit by INAA was 0.012 μg of As^III^/g. Urine sample collection and analysis were the same as those described by [25]. Briefly, morning urine samples were collected from participants on three consecutive days and shipped on dry ice to the University of Alberta, Edmonton, AB, Canada for analysis. Inductively coupled mass spectrometry methods were used to measure urinary arsenic, with a detection limit of 0.5 μg of As^III^/L.

### 2.4. Blood Collection, RNA Isolation, and cDNA Synthesis

Blood samples were collected in PAXgene blood RNA tubes (Qiagen, Valencia, CA, USA) and then stored at −40 °C. The blood samples were transported to the U.S. A via air on blue ice and stored at −80 °C until RNA isolation. Total RNA was isolated using a PAXgene blood RNA kit according to the manufacturer’s instructions. RNA was further subjected to DNase I treatment using the Turbo DNA-free Kit following manufacturer’s instructions (Applied Biosystems, Foster City, CA, USA). RNA quality was assessed by analysis with Agilent Bioanalyer 2100 Nono chip.

### 2.5. cDNA Synthesis

Total RNA was reverse-transcribed to cDNA by using High-Capacity cDNA Reverse Transcription Kits (Applied Biosystems, CA, USA). The reaction mix was consisted of 2.5 U/μL of reverse transcriptase, 1.0 U/μL of RNase inhibitor, 0.4-mM dNTP, 0.2-μM random primers, and 2 μg RNA in 50 μL total volume. The reaction mixture was incubated at 25 °C for 10 min and then at 37 °C for 120 min. Finally, the mixture was heated at 85 °C for 5 s.

### 2.6. TaqMan Low-Density Array

To identify altered gene expression in blood cells, we used a TLDA to examine the expression of 46 human genes. The 46 genes we selected occurred in several functional groupings (Table 1) that represented proposed mode-of-actions of arsenic based on human epidemiology, as well as animal and *in vitro* studies. For the present study, the TaqMan low-density array card was configured in to eight identical 48 genes set (duplicate per assay). Each set of genes contained two endogenous control genes, β-actin and 18s RNA. The cDNA (5 μL) was mixed with 45 μL of H_2_O and 50 μL of 2X TaqMan Universal PCR Mix (Applied Biosystems, Foster City, CA). Each sample (100 μL) was loaded into a port of the micro-fluid card and run on an ABI 7900HT System (ABI, CA) for 2 min at 50 °C, 10 min at 95 °C, followed by 40 cycles for 15 s at 97 °C and 1 min at 60 °C.

### 2.7. *In Vitro* Studies

The human cardiomyocyte cell line (AC16) was provided by one of us (M. Davidson) and were cultured in Dulbecco’s Modified Eagle’s/F12 medium supplemented with 12.5% fetal bovine serum (Invitrogen, Carlsbad, CA, USA), penicillin (100 U/mL), and streptomycin (100 μg/mL) and maintained at 37 °C in 95% air and 5% CO_2_. The MTT cell-proliferation assay kit (Roche, Indianapolis, IN, USA) was used to assess cell viability. Briefly, 1X 10^4^ AC16 cells were seeded in 96-well plates in 0.2 mL of medium. After 24 h of incubation, sodium arsenite was added to the cultures at final concentrations of 0.1 to 5.0 μM, and cells were incubated for 3 days. Cell viability was measured at 560 nm and expressed as a percentage of controls. The assay was conducted in triplicate wells for each treatment, and a total of 3 experiments were conducted for all samples.

### 2.8. Arsenite Treatment and RNA Isolation

After incubating the seeded cells for 24 h, the cells were treated with sodium arsenite (Sigma, St. Louis, MO, USA) at concentrations of 0 to 2.5 μM for 72 h. Total RNA was isolated from the control and the arsenite-treated cells using the RNeasy RNA Isolation Kit (Qiagen, CA, USA). cDNA synthesis and the TILDA were conducted as described above; each sample was tested in duplicate.

### 2.9. Data Analysis

The TLDA data were analyzed by SDS vers 2.2.2 software (ABI). Threshold cycle (Ct) data for all target genes and control gene 18s RNA were used to calculate ΔCt values [ΔCt = Ct (target gene) – Ct (18s RNA)]. Then, ΔΔCt values were calculated by subtracting the calibrator (control) from the ΔCt values of each target. Multiple linear regression models were used to evaluate the relationship between gene expression (ΔCt) and nail, water, and urine arsenic levels. Because arsenic measures were highly right-skewed, they were log_10_-transformed prior to analysis. The dependent variable was gene expression as measured by ΔCt value, and the independent variable of interest was the log-transformed arsenic measure. Log transformation did not improve the skew of ΔCt measures, which tended to be fairly symmetric.

Three types of models were constructed: an unadjusted model; a model that included variables to adjust for age and sex; and a model adjusting for age, body mass index, sex, alcohol use, pesticide exposure in the past five years, and education level. Because adjusting for factors other than age and sex did little to change the relationship between arsenic density and gene expression, results adjusting for age and sex were reported. Slope coefficients shown in Table 3 can be interpreted as the change in gene expression (ΔCt) associated with a 1-log_10_ increase in the water, urine, or nail arsenic levels. Non-linearity in the relationship between arsenic and gene expression was evaluated by fitting a two-degree fractional polynomial model and comparing it with a linear model using a deviance difference test [26]. Statistical analyses were conducted using Stata SE version 10.1 (Stata Corporation, College Station, TX, USA, 2008).

## 3. Results

### 3.1. Study Subjects

Demographic information and pesticide use related to the study subjects are shown in Table 2. All subjects were non-smokers. Most (79%) of the 159 study subjects, including 48 males and 111 females, were farmers. The nail arsenic concentrations ranged from 0.24 to 63.14 μg/g. Water arsenic concentrations ranged from non-detectable to 826 mg/L. The average years of arsenic exposure for the study subjects was 13 ± 6 (SD) years.

### 3.2. Variability of Gene Expression Data from TLDA

**Figure 1.**
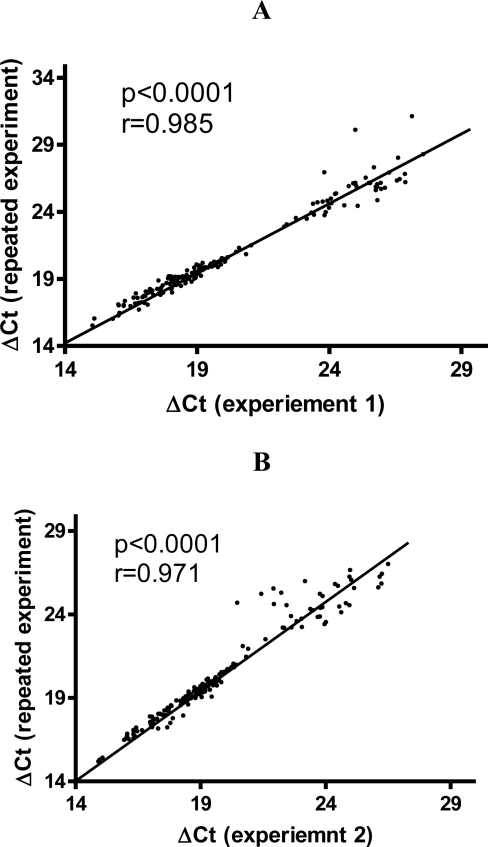
Viability of cardiomyocytes exposed to sodium arsenite. AC16 cells were seeded in a 96-well plate and exposed for three days to various concentrations of sodium arsenite. Cell viability was determined by the MTT assay. Data shown are mean ± SD from three independent experiments.

To assess the reproducibility of the TILDA, sixteen samples of cDNAs from human blood were run repeatedly. ΔCt (The Ct values normalized to loading control) for experiment 1 were plotted against ΔCt for repeated TLDA experiments. The data showed a small variation with high correlation coefficients (0.985 and 0.971) (Figures 1A,B). The data showed that the ΔCts were reproducible.

### 3.3. Arsenic-Altered Gene Expression in the Blood

The TLDA showed that the majority of selected genes were expressed in the blood, most of them with Cts < 30. However, *hTERT* and *CANC1* were under the detectable limit (Ct > 37) and, thus, were excluded from further analysis. A linear regression was employed to determine the significance of association of expression with water arsenic levels. The data from *KCNA5* and *TNFα* expression showed a positive association with water arsenic exposure (Table 3). Furthermore, the expression level of KCNA5 was also associated with nail arsenic concentrations, whereas that of TNF-α was also associated with urine arsenic concentrations.

In addition to *KCNA5* and *TNF*-α, the altered expressions of nine other genes were associated with urinary arsenic concentrations (Table 3). The associated molecular functions for these 11 gene products included stress response, inflammation, apoptosis, DNA methylation, DNA repair, and the NF-κB signal transduction pathway. TNF-α is the prototypic pro-inflammatory cytokine and master regulator of cell apoptosis, inflammation, and tumorigenesis. TNF-α is an important constituent of the NF-kB signal pathway. There was no evidence of a significant (*p* < 0.05) non-linear relationship between water, nail, or urine arsenic levels and gene expression, and in all cases a 2-degree fractional polynomial model was not an improvement over a linear regression model.

### 3.4. Cardiomyocytes Treated with Arsenite *In Vitro*

**Figure 2.**
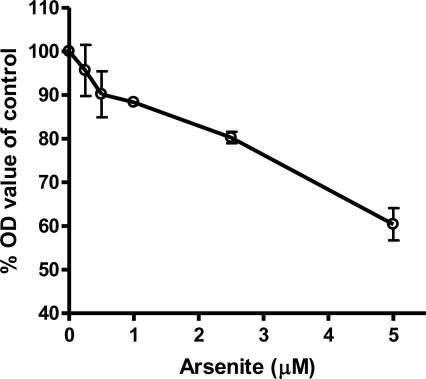
Correlation plot of mean ΔCt values from repeated TLDA experiments. Panels A and B show representative ΔCt values obtained from repeated experiment with two different samples. The ΔCt (The Ct values normalized to loading control) for experiment 1 was plotted against the ΔCt for repeated TILDA runs. Linear regression analysis was used to determined Pearson correlation coefficient (r) and *p* values.

**Figure 3.**
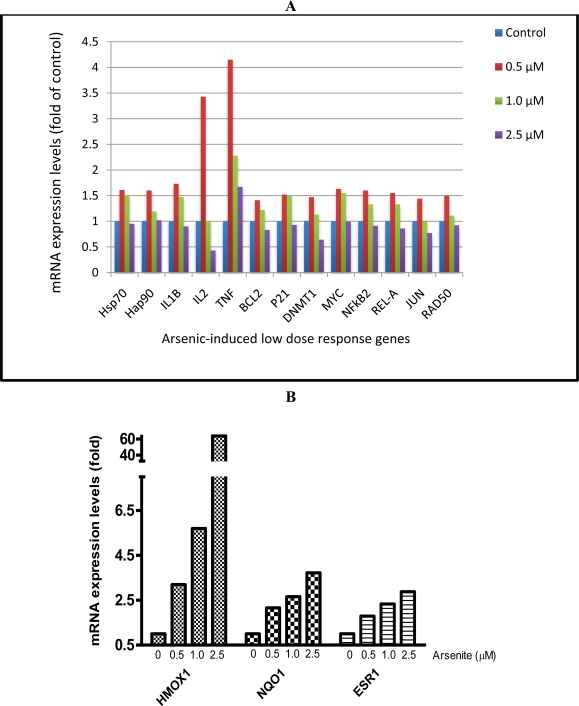

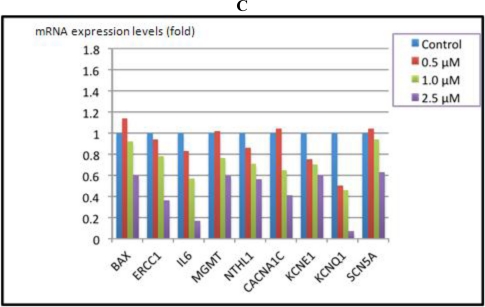
Gene expression analysis of cardiomyocyte AC16 cells exposed to sodium arsenite for 72 h as determined by a TLDA.

Results from the MTT assay showed that the viability of AC16 cells after arsenic treatment decreased as arsenite concentrations increased (Figure 2). For the mRNA levels of 46 genes, three different patterns of response were observed. The expression of one group of genes was increased at low concentrations of arsenic (0.5 and 1 μM) but decreased at higher concentrations (>2.5 μM, Figure 3A). These included the genes associated with heat shock proteins, inflammatory molecules, apoptosis, DNA repair, and the NFkB pathways (Figure 3A). A second group, including the genes *HMOX1*, *NQO1* and *ESR1*, showed increased expression relative to increased arsenic concentrations up to 5 μM (Figure 3B). A third group showed down-regulated gene expression relative to arsenic concentration, and these included the ion channel genes *CACNA1C, KCNE1, KCNQ1* and *SCDA5*; the DNA repair genes *ERCC1* and *NTHL1*; and the cytokine gene IL6 (Figure 3C).

Values in Figure 3 are fold changes of expression in treated cells relative to control cells. The ΔCt values of untreated cells were designated as calibrators. The relative quantity (RQ) was calculated by the equation RQ = 2^−ΔΔCt^. (A) Up-regulated gene expression at low concentrations of arsenic but down-regulated expression at high concentrations (2.5 μM). (B) Up-regulated gene expression relative to increasing concentrations of arsenic. (C) Down-regulated gene expression relative to increasing concentrations of arsenic.

## 4. Discussion

### 4.1. Changes in Gene Expression in Blood Associated with Chronic Arsenic Exposure

In this study, we investigated the molecular responses to arsenic exposure in humans and cultured cardiomyocytes using a novel TLDA in order to better understand the mode of action of arsenic. In blood, we found that alterations in expression of 11 genes, especially those associated with stress response, DNA repair, DNA methylation, the NF-κB signaling pathway, and iron channels, were positively associated with arsenic exposure. Urinary arsenic concentrations were associated with altered expression of 10 genes, 8 of which were associated only with urinary arsenic levels. Expression of 3 genes was associated with binary combinations of the 3 exposure assessments (Table 3). Based on the TLDA we designed, urinary arsenic was the most robust measure of exposure. Considering all the exposure estimates, expression of *TNF-α* was associated with both water and urinary concentrations of arsenic, that of *POLB* was associated with nail and urinary levels, and that of *KCNA5* was associated with water and nail levels (and was borderline significant with urinary arsenic). Because altered expression of these genes was associated with 2 of the 3 exposure assessments, we view that arsenic exposure clearly played a role in the altered expression of *KCNA5*, *TNF-α*, and *POLB* as assessed by the TLDA in this study.

Our finding that altered gene expression was associated most frequently with urinary arsenic levels is interesting in light of the finding that urinary arsenic levels were a stronger predictor of skin lesions than arsenic levels in drinking water [27,28]. In addition, chromosomal aberrations have been shown to be associated with urinary arsenic [29]. Urinary arsenic receives input from external sources and tissue storage and likely reflects the body burden of arsenic. Consequently, altered gene expression associated with urinary arsenic may reflect current arsenic exposure levels.

Expression of *KCNA5* influences various cell functions, such as cell migration, proliferation and apoptosis. Over-expression of human *KCNA5* in pulmonary artery smooth muscle cells induced membrane hyperpolarization and increased cell apoptosis [30]. Down-regulation of *KCNA5* expression by an antisense oligonucleotide resulted in membrane depolarization [31]. *KCNA5* expression occurs in response to oxidative stress mediated by the Sp1 transcription factor [32].

TNF-α is a cytokine involved in cell proliferation, differentiation, and apoptosis. Induction of TNF-α by arsenic exposure has been observed consistently both *in vitro* and *in vivo* [33,34]. Over-expression of *TNF-α* and *GM-CSF* has been associated with neoplastic transformation in the skin. *TGF*-α transgenic mice exhibited keratinocyte hyper-proliferation and tumors in the epithelium [35]. Our PCR array profiling showed that *TNF*-α expression was positively associated with chronic arsenic exposure, indicating that this inflammation cytokine is involved in arsenic toxicity and possibly carcinogenesis. Some TNFs may induce both apoptotic and anti-apoptotic pathways, the increased expression of TNF-α, along with BCL2 and BAX, strongly indicate the activation of the anti-apoptotic/NF-κB/BCL2 pathway in the subjects exposed to environmental arsenic.

Expression of *JUN* and *MAF* was positively associated with urinary arsenic levels in this study. The coordinated induction of these two genes suggests that the *Nrf2* pathway plays a crucial role in the cellular defense against arsenic-induced oxidative stress, which has been reported to be involved in the toxicity induced by low-dose arsenic exposure [36]. The Nrf2 pathway plays an important role by mediating an antioxidant response against the adverse effects of excess ROS production generated by arsenic metabolites [37]. The transcription factor Nrf2 is a central regulator in the activation of many genes related to antioxidant response, such as glutathione S-transferase (*GST*), *NAD(P)H*, quinine reductases (*NQO1*), heat shock protein, and heme oxygenase 1 (*HO-1*). Although we did not have *GGT1* or *NFKBIE* on our array, their expression levels in lymphoblasts may be biomarkers of susceptibility arsenic toxicity [38].

The small Maf basic leucine zipper protein has emerged as a crucial regulator of mammalian gene expression. During oxidative stress, Nrf2 heterodimerizes with small Maf and binds antioxidant response element (ARE) sequences, thereby transcriptionally activating ARE [37]. The activator protein (AP-1) transcription factor is a protein complex consisting c-Jun and c-Fos, which are involved in the regulation of many different kind of cellular processes, including proliferation and survival, differentiation, and transformation. Consistent with this is the finding that expression of *c-Jun* and *c-Fos* was up-regulated in human GM847 fibroblast cells exposed to arsenic [39]. Our finding of up-regulated expression of *c-Jun* and *Maf* in humans exposed chronically to arsenic suggests that arsenic may alter *AP-1* and *NF*-κ*b* activity. The NFκB signaling pathway is involved in the immune response, inflammation, and apoptosis, and the inflammation/NF-κB signaling pathway was reported to be activated in infants born to arsenic-exposed mothers [15]. Our data and that of others [15,40] suggest that NFκB activation induced by arsenic exposure may play an important role in arsenic toxicity in humans.

Arsenic exposure is associated with a variety of diseases, including cancer, cardiovascular disease, and diabetes [1,8,41]. The mechanisms underlying the carcinogenicity of arsenic are varied; however, oxidative stress is a likely mode of action for arsenic [1,3]. Arsenic metabolites generate ROS, resulting in damage to DNA and other macromolecules. For example, ROS-generating metabolites of arsenic can induce DNA breaks, resulting in chromosomal mutations [42]. The association of arsenic exposure with increased gene expression of nucleotide excision repair, DNA methylation, and oxidative DNA repair pathways observed in this study is consistent with this mechanism of arsenic carcinogenicity. As discussed below, oxidative stress may also play a role in the cardiovascular diseases associated with arsenic exposure, further supporting a role for the genes identified here in arsenic-associated disease.

### 4.2. Changes in Gene Expression in Arsenic-Exposed Cardiomyocytes

Several reviews have concluded that arsenic exposure is associated with cardiovascular disease [8,41,43]. The specific diseases include carotid atherosclerosis, reduced microcirculation, prolonged QT interval and increased QT dispersion, hypertension, coronary artery disease, and cerebral infarction. Epidemiology studies support the view that chronic arsenic exposure is an independent risk factor for cardiovascular disease. Our previous work has demonstrated a clear relationship between chronic arsenic exposure and QT interval prolongation [5].

The mechanistic basis for this collection of arsenic-associated health effects appears to involve a role for oxidative stress and activated immune response pathways, indicating a pro-hyperinflammatory condition [8]. In arsenic-exposed mice, the arterial wall is inflamed, and liver sinusoidal endothelium differentiates into a continuous endothelium that limits nutrient exchange and waste elimination. Thus, current models focus on altered signaling by arsenic, resulting in inflammation of liver and heart arteries [8].

To our knowledge, our study is the first to expose heart cells (cardiomyocytes) to arsenic *in vitro*. We found that 25 of the 46 genes assessed by our TLDA exhibited altered expression after exposure of cardiomyocytes to arsenic (Figure 3). Most interesting was that these affected genes fell into three categories: (1) those whose expression was up-regulated at low concentrations of arsenic but down-regulated at high concentrations of arsenic (Figure 3A), those whose expression was up-regulated with increasing concentrations of arsenic (Figure 3B), and those whose expression were down-regulated with increasing concentrations of arsenic (Figure 3C).

Various studies have noted consistently that, for some genes, arsenic induces expression at low doses but reduces expression at high doses *in vitro* and *in vivo*. In human keratinocytes, sodium arsenite induced apurinic/apyrimidinic endonuclease, DNA polymerase β, DNA ligase, and telomerase up to 1 μM, but down-regulated these genes at concentration >1 μM [39,44,45]. The sub-micromolar concentrations of sodium arsenite used in our study are similar to the levels of arsenic found in the blood of subjects exposed chronically to arsenic in Inner Mongolia [46]. These results suggest that low doses of arsenic trigger an adaptive response that alleviates the adverse effects of arsenic cytotoxicity and oxidative stress. Some of this response could protect against cancer by elevated DNA repair capacity. Other responses, such as increased cell proliferation, or inhibited apoptosis, may be cytoprotective, but could also be procarcinogenic by allowing mutant cells to survive.

Transcriptional regulation plays a key role in the regulation of potassium channel genes involved in cardiac muscle function and accounts for some of the variation of the elecrtro-physiological phenotype of myocytes [47]. Therefore, expression of *KCNA5* may have been altered by arsenic in the cardiomyocytes, but because of the low levels of this mRNA in our cells, we could not confirm such a change. However, we observed transcriptional changes for several iron-channel genes, including *KCNE1, KCNH2,* and *CADA1C* in cardiomyocytes treated with arsenite. Alterations in such genes could play a role in the cardiac arrhythmias associated with arsenic exposure [5,48].

As noted earlier, epidemiological studies have identified arsenic exposure as a causative agent for diabetes [5,41,43]. Although we did not design our TLDA to assess genes potentially involved in arsenic-associated diabetes, recent studies [49,50] suggest that the arsenic-associated decrease in insulin secretion found in rodent models may be partially responsible for the altered cell division and proliferation associated with arsenic exposure. *In vitro* studies found that free Ca^(2+)^ oscillations needed for glucose-stimulated insulin secretion were abated in the presence of subchronic, low arsenite concentrations [50]. Thus, it is interesting that we found that arsenic reduced expression of a variety of ion channel genes, including the calcium channel, voltage-dependent, L type alpha 1C subunit gene *CACNA1C* in cardiomyocytes (Figure 3C). Although it is difficult to make firm conclusions and direct comparisons of gene expression in cardiomyocytes and blood due to the nature of the study design and differences in absorption and metabolismwe found that five genes showed altered expression both in blood and in the treated cardiomyocytes: *BAX, DNMT1, JUN, NTHL1,* and *RAD50*.

## 5. Conclusions

In conclusion, this study shows that the TLDA is a sensitive and reproducible technique for quantifying expression of multiple genes. Our finding in the blood of arsenic-exposed subjects of the simultaneous increase in gene expression involved in antioxidant response, inflammatory response, DNA repair, apoptosis, DNA methylation, and iron channels, suggests an integrative mode of action of arsenic in humans. Oxidative stress, inflammatory response, and altered ion-channel function may underlay aspects of the cancer, cardiovascular disease, and diabetes associated with chronic arsenic exposure. A recent study has also identified the importance of epigenetic changes in people with arsenicosis [51]. Further studies involving specific cell types are needed to identify the complete set of pathways affected by arsenic that result in the array of seemingly unrelated diseases.

## Figures and Tables

**Table 1.** Genes analyzed by TLDA.

**Gene symbol and assay ID**	**Functional group**	**Gene description**
ACTB-Hs00242273_m1	Endogenous control	beta actin
18S-Hs99999901_s1		18S Ribosomal RNA

HMOX1-Hs00157965_m1	Nrf2 pathway	
KEAP1-Hs00202227_m1		kelch-like ECH-associated protein 1
MAF-Hs00193519_m1		
MT1A-Hs00831826_s1		
NQO1-Hs00168547_m1		

HSPA1A-Hs00359163_s1	Heat shock protein	
HSP90AA1-Hs00743767_sH		

BAX-Hs00180269_m1	Apoptosis	
BCL2-Hs00153350_m1		

IL1B-Hs00174097_m1	Inflammation	
IL2-Hs00174114_m1		
IL6-Hs00174131_m1		
TNF-Hs00174128_m1		

CHUK-Hs00175141_m1	NF-κB pathway	
NFE2L2-Hs00232352_m1		nuclear factor (erythroid-derived 2)-like 2
NFKB1-Hs00231653_m1		nuclear factor in B-cells 1 (p105)
NFKB2-Hs00174517_m1		nuclear factor in B-cells 2 (p49/p100)
RELB-Hs00232399_m1		
RELA-Hs00153294_m1		

CDKN1A-Hs00355782_m1	Cell proliferation	
CCND1-Hs00277039_m1		
ESR1-Hs00174860_m1		
FOS-Hs00170630_m1		
JUN-Hs99999141_s1		
MYC-Hs00153408_m1		
MSH2-Hs00179887_m1		
NRAS-Hs00180035_m1		
TERT-Hs00162669_m1		

DNMT1-Hs00154749_m1	DNA methylation	DNA (cytosine-5-)-methyltransferase 1
DNMT3A-Hs00173377_m1		

ERCC1-Hs00157415_m1	DNA repair	
MGMT-Hs00172470_m1		
NTHL1-Hs00267385_m1		
OGG1-Hs00213454_m1		
POLB-Hs00160263_m1		

DNMT1-Hs00154749_m1	DNA methylation	DNA (cytosine-5-)-methyltransferase 1
RAD50-Hs00194871_m1		
TP53-Hs00153349_m1		

CACNA1C-Hs00167681_m1	Ion channel	calcium channel, voltage-dependent, L type, alpha 1C subunit
SCN5A-Hs00165693_m1		sodium channel, voltage-gated, type V, alpha (long QT syndrome 3)
KCNJ2-Hs00265315_m1		potassium inwardly-rectifying channel, subfamily J, member 2
KCNA5-Hs00266898_s1		potassium voltage-gated channel, shaker-related subfamily, member 5
KCND3-Hs00542597_m1		potassium voltage-gated channel, Shal-related subfamily, member 3
KCNE1-Hs00264799_s1		potassium voltage-gated channel, Isk-related family, member 1
KCNQ1-Hs00165003_m1		potassium voltage-gated channel, KQT-like subfamily, member 1
KCNH2-Hs00165120_m1		potassium voltage-gated channel, subfamily H (eag-related), member 2
KCNE2-Hs00270822_s1		potassium voltage-gated channel, Isk-related family, member 2

**Table 2.** Selected characteristics of study population.

**Variables**	**Number of subjects**	**Percent**
*Gender*		
Male	48	30.2
Female	111	69.8
*Age*		
11–18	49	30.8
19–49	93	58.5
>50	17	10.7
*Occupation*		
Farmer	110	69.6
Other	48	30.4
*Education*		
None	24	15.2
Elementary	50	31.6
Jr high school	76	48.1
High school	8	5.1
*Meat frequency*		
Often	159	100
*Vegetable frequency*		
Occasionally	1	0.6
Often	158	99.4
*Use of pesticides in past 5 years*		
Yes	45	28.3
No	114	71.7
*Alcohol use 2 x/week*		
No	114	95.6
Yes	7	4.4
*Eat fish frequency*		
Never	1	0.6
Occasionally	146	91.8
Often	12	7.5

**Table 3.** Association of gene expression with water, nail, or urine arsenic levels^a^.

**Gene**	**Function group**	**Water arsenic**		**Nail arsenic**		**Urine arsenic**	

		**Slope**	**p value^b^**	**Slope**	**p value**	**Slope**	**p value**
*KCNA5*	Ion channel	0.343	0.031	0.547	0.043	0.189	0.078
*TNF-α*	Inflammation	0.178	0.043			0.146	0.013
*POLB*	DNA repair			0.306	0.049	0.135	0.028
*MAF*	Nrf2 pathway					0.137	0.038
*BCL2*	Apoptosis					0.131	0.033
*BAX*	Apoptosis					0.117	0.038
*DNMT1*	DNA methylation					0.122	0.031
*HSP1*	Heat shock protein					0.105	0.054
*JUN*	Signal transduction					0.149	0.023
*NTHL1*	DNA repair					0.132	0.034
*RAD50*	DNA repair					0.113	0.050

a Only those slopes and *p*-values with significant association with arsenic (*p* < 0.05) are shown;

b N = 159, *p*-values were age and sex adjusted.

## References

[1] International Agency for Research on Cancer (IARC) (2004). IARC Monographs on the Evaluation of Carcinogenic Risks to Humans. Some Drinking-Water Disinfectants and Contaminants, Including Arsenic.

[2] Tapio S, Grosche B (2006). Arsenic in the aetiology of cancer. Mutat. Res.

[3] Straif K, Benbrahim-Tallaa L, Baan R, Grosse Y, Secretan B, El Ghissassi F, Bouvard V, Guha N, Freeman C, Galichet L (2009). A review of human carcinogens—Part C: Metals, arsenic, dusts, and fibres. Lancet Oncol.

[4] Rossman TG, Uddin AN, Burns FJ (2004). Evidence that arsenite acts as a cocarcinogen in skin cancer. Toxicol. Appl. Pharmacol.

[5] Mumford JL, Wu K, Xia Y, Kwok R, Yang Z, Foster J, Sanders WE (2007). Chronic arsenic exposure and cardiac repolarization abnormalities with QT interval prolongation in a population-based study. Environ. Health Perspect.

[6] Westervelt P, Brown RA, Adkins DR, Khoury H, Curtin P, Hurd D, Luger SM, Ma MK, Ley TJ, DiPersio JF (2001). Sudden death among patients with acute promyelocytic leukemia treated with arsenic trioxide. Blood.

[7] Sanz MA, Grimwade D, Tallman MS, Lowenberg B, Fenaux P, Estey EH, Naoe T, Lengfelder E, Bűchner T, Dőhner H, Burnett AK, Lo-Coco F (2009). Management of acute promyelocytic leukemia: recommendations from an expert panel on behalf of the European LeukemiaNet. Blood.

[8] States JC, Srivastava S, Chen Y, Barchowsky A (2009). Arsenic and cardiovascular disease. Toxicol. Sci.

[9] Wade TJ, Xia Y, Wu K, Li Y, Ning Z, Le XC, Lu X, Feng Y, He X, Mumford JL (2009). Increased mortality associated with well-water arsenic exposure in Inner Mongolia, China. Int. J. Environ. Res. Public Health.

[10] Wang CH, Chen CL, Hsiao CK, Chiang FT, Hsu LI, Chiou HY, Hsueh YM, Wu MM, Chen CJ (2009). Increased risk of QT prolongation associated with atherosclerotic diseases in arseniasis-endemic area in southwestern coast of Taiwan. Toxicol. Appl. Pharmacol.

[11] Mordukhovich I, Wright RO, Amarasiriwardena C, Baja E, Baccarelli A, Suh H, Sparrow D, Vokonas P, Schwartz J (2009). Association between low-level environmental arsenic exposure and QT interval duration in a general population study. Am. J. Epidemiol.

[12] Liu J, Kadiiska MB, Liu Y, Lu T, Qu W, Waalkes MP (2001). Stress-related gene expression in mice treated with inorganic arsenicals. Toxicol. Sci.

[13] Rea MA, Gregg JP, Qin Q, Phillips MA, Rice RH (2003). Global alteration of gene expression in human keratinocytes by inorganic arsenic. Carcinogenesis.

[14] Argos M, Kibriya MG, Parvez F, Jasmine F, Rakibuz-Zaman M, Ahsan H (2006). Gene expression profiles in peripheral lymphocytes by arsenic exposure and skin lesion status in a Bangladeshi population. Cancer Epidemiol. Biomarkers Prev.

[15] Fry RC, Navasumrit P, Valiathan C, Svensson JP, Hogan BJ, Luo M, Bhattacharya S, Kandjanapa K, Soontararuks S, Nookabkaew S (2007). Activation of inflammation/NF-κB signaling in infants born to arsenic-exposed mothers. PLoS Genet.

[16] Ghosh P, Banerjee M, Giri AK, Ray K (2008). Toxcogenomics of arsenic: Classical ideas and recent advances. Mutat. Res.

[17] Abruzzo LV, Lee KY, Fuller A, Silverman A, Keating MJ, Medeiros LJ, Coombes KR (2005). Validation of oligonucleotide microarray data using microfluidic low-density arrays: A new statistical method to normalize real-time RT-PCR data. Biotechniques.

[18] Goulter AB, Harmer DW, Clark KLO (2006). Evaluation of low density array technology for quantitative parallel measurement of multiple genes in human tissue. BMC Genomics.

[19] Ma HZ, Xia YJ, Wu KG, Sun TZ, Mumford JL, Abernathy WR, Calderon CO, Chappell R (1999). Human exposure to arsenic and health effects in Bayingnormen, Inner Mongolia. Arsenic Exposure and Health Effects.

[20] Mo J, Xia Y, Wade TJ, Schmitt M, Le XC, Dang R, Mumford JL (2006). Chronic arsenic exposure and oxidative stress: OGG1 expression and arsenic exposure, nail selenium, and skin hyperkeratosis in Inner Mongolia. Environ. Health Perspect.

[21] World Medical Association (1989). Declaration of Helsinki.

[22] Gong Z, Lu X, Watt C, Wen B, He B, Mumford J, Ning Z, Xia Y, Le XC (2006). Speciation analysis of arsenic in groundwater from Inner Mongolia with an emphasis on acid-leachable particulate arsenic. Anal. Chim. Acta.

[23] Schmitt MT, Schreinemachers D, Wu K, Ning Z, Zhao B, Le XC, Mumford JL (2005). Human nails as a biomarker of arsenic exposure from well water in Inner Mongolia: Comparing atomic fluorescence spectrometry and neutron activation analysis. Biomarkers.

[24] Heydorn K (1984). Neutron Activation Analysis for Clinical Trace Element Research.

[25] Otto D, Xia Y, Li Y, Wu K, He L, Telech J, Hundell H, Prah J, Mumford J, Wade T (2007). Neurosensory effects of chronic human exposure to arsenic associated with body burden and environmental measures. Hum. Exp. Toxicol.

[26] Royston P, Ambler G, Sauerbrei W (1999). The use of fractional polynomials to model continuous risk variables in epidemiology. Int. J. Epidemiol.

[27] Watanabe C, Inaoka T, Kadono T, Nagano M, Nakamura S, Ushijima K, Murayama N, Miyazaki K, Ohtsuka R (2001). Males in rural Bangladeshi communities are more susceptible to chronic arsenic poisoning than females: Analyses based on urinary arsenic. Environ. Health Perspect.

[28] Ahsan H, Perrin M, Rahman A, Parvez F, Stute M, Zheng Y, Milton AH, Brandt-Rauf P, van Geen A, Graziano J (2000). Associations between drinking water and urinary arsenic levels and skin lesions in Bangladesh. J. Occup. Environ. Med.

[29] Maki-Paakkanen J, Kurttio P, Paldy A, Pekkanen J (1998). Association between the clastogenic effect in peripheral lymphocytes and human exposure to arsenic through drinking water. Environ. Mol. Mutagen.

[30] Bevnova EE, Platoshyn O, Zhang S, Yuan JX (2004). Overexpression of human KCNA5 increases IK V and enhances apoptosis. Am. J. Physiol. Cell Physiol.

[31] Nattel S, Bourne G, Talajic M (1997). Insights into mechanisms of antiarrythmic drug action from experimental models of atrial fibrillation. J. Cardiovasc. Electrophysiol.

[32] Fountain SJ, Cheong A, Li J, Dondas NY, Zeng F, Wood IC, Beech DJ (2007). K9v1.5 potassium channel gene regulation by Sp1 transcription factor and oxidative stress. Am. J. Physiol. Heart Circ. Physiol.

[33] Germolec DR, Spalding J, Boorman GA, Wilmer JL, Yoshida T, Simeonova PP, Bruccoleri A, Kayama F, Gaido K, Tennant R (1997). Arsenic can mediate skin neoplasia by chronic stimulation of keratinocyte-derived growth factors. Mutat. Res.

[34] Yih LH, Peck K, Lee TC (2002). Changes in gene expression profiles of human fibroblasts in response to sodium arsenite treatment. Carcinogenesis.

[35] Simeonova PP, Luster MI (2000). Mechanisms of arsenic carcinogenicity: Genetic or epigenetic mechanisms?. J. Environ. Pathol. Toxicol. Oncol.

[36] Schoen A, Beck B, Sharma R, Dube E (2004). Arsenic toxicity at low doses: Epidemiological and mode of action considerations. Toxicol. Appl. Pharmacol.

[37] Itoh K, Chiba T, Takahashi S, Ishii T, Igarashi K, Katoh Y, Oyake T, Hayashi N, Satoh K, Hatayama I (1997). An Nrf2/small Maf heterodimer mediates the induction of phase II detoxifying enzyme genes through antioxidant response elements. Biochem. Biophys. Res. Commun.

[38] Komissarova EV, Li P, Uddin AN, Chen X, Nadas A, Rossman TG (2008). Gene expression levels in normal human lymphoblasts with variable sensitivities to arsenite: Identification of GGT1 and NFKBIE expression levels as possible biomarkers of susceptibility. Toxicol. Appl. Pharmacol.

[39] Hu Y, Jin X, Snow ET (2002). Effect of arsenic on transcription factor AP-1 and NF-kappaB DNA binding activity and related gene expression. Toxicol. Lett.

[40] Liu Q, Zhang H, Smeester L, Zou F, Kesic M, Jaspers I, Pi J, Fry RC (2010). The NRF2-mediated oxidative stress response pathway is associated with tumor cell resistance to arsenic trioxide across the NCI-60 panel. BMC Med. Genomics.

[41] Navas-Acien A, Sharrett AR, Silbergeld EK, Schwartz BS, Nachman KE, Burke TA, Guallar E (2005). Arsenic exposure and cardiovascular disease: A systematic review of the epidemiologic evidence. Am. J. Epidemiol.

[42] Kligerman AD, Doerr CL, Tennant AH, Harrington-Brock K, Allen JW, Winkfield E, Poorman-Allen P, Kundu B, Funasaka K, Roop BC, Mass MJ, DeMarini DM (2003). Methylated trivalent arsenicals as candidate ultimate genotoxic forms of arsenic: induction of chromosomal mutations but not gene mutations. Environ. Mol. Mutagen.

[43] Wang CH, Hsiao CK, Chen CL, Hsu LI, Chiou HY, Chen SY, Hsueh YM, Wu MM, Chen CJ (2007). A review of the epidemiologic literature on the role of environmental arsenic exposure and cardiovascular diseases. Toxicol. Appl. Pharmacol.

[44] Sykora P, Snow ET (2008). Modulation of DNA polymerase beta-dependent base excision repair in cultured human cells after low dose exposure to arsenite. Toxicol. Appl. Pharmacol.

[45] Zhang TC, Schmitt MT, Mumford JL (2003). Effects of arsenic on telomerase and telomeres in relation to cell proliferation and apoptosis in human keratinocytes and leukemia cells *in vitro*. Carcinogenesis.

[46] Pi J, Yamauchi H, Kumagai Y, Sun G, Yoshida T, Aikawa H, Hopenhayn-Rich C, Shimojo N (2002). Evidence for induction of oxidative stress caused by chronic exposure of Chinese residents to arsenic contained in drinking water. Environ. Health Perspect.

[47] Dixon JE, McKinnon D (1994). Quantitative analysis of potassium channel mRNA expression in atrial and ventricular muscle of rats. Circ. Res.

[48] Wan X, Dennis AT, Obejero-Paz C, Overholt JL, Heredia-Moya J, Kirk KL, Ficker E (2011). Oxidative inactivation of the lipid phosphatase phosphatase and tensin homolog on chromosome ten (PTEN) as a novel mechanism of acquired long QT syndrome. J. Biol. Chem.

[49] Diaz-Villaseñor A, Burns AL, Hiriart M, Cebrián ME, Ostrosky-Wegman P (2007). Arsenic-induced alteration in the expression of genes related to type 2 diabetes mellitus. Toxicol. Appl. Pharmacol.

[50] Diaz-Villaseñor A, Hiriart M, Cebrián ME, Zacarías-Castillo R, Ostrosky-Wegman P (2008). The activity of calpains in lymphocytes is glucose-dependent and is decreased in diabetic patients. Blood Cells Mol. Dis.

[51] Smeester L, Rager JE, Bailey KA, Guan X, Smith N, García-Vargas G, Del Razo LM, Drobná Z, Kelkar H, Stýblo M, Fry RC (2011). Epigenetic changes in individuals with arsenicosis. Chem. Res. Toxicol.

